# A methodological protocol for selecting and quantifying low-value prescribing practices in routinely collected data: an Australian case study

**DOI:** 10.1186/s13012-017-0585-9

**Published:** 2017-05-03

**Authors:** Jonathan Brett, Adam G. Elshaug, R. Sacha Bhatia, Kelsey Chalmers, Tim Badgery-Parker, Sallie-Anne Pearson

**Affiliations:** 1 Medicines Policy Research Unit, Centre for Big Data Research in Health, University of New South Wales, Level 1, AGSM Building (G27), Sydney, NSW 2052 Australia; 2 Menzies Centre for Health Policy, School of Public Health, University of Sydney, Level 6 The Hub, Charles Perkins Centre D17, Sydney, NSW 2006 Australia; 3 Department of Medicine, University of Toronto, Toronto, Canada; 4 Institute for Health System Solutions and Virtual Care, Women’s College Hospital, Toronto, Canada

**Keywords:** Routinely collected data, Prescribing, Medicines, Low-value care, Protocol

## Abstract

**Background:**

Growing imperatives for safety, quality and responsible resource allocation have prompted renewed efforts to identify and quantify harmful or wasteful (low-value) medical practices such as test ordering, procedures and prescribing. Quantifying these practices at a population level using routinely collected health data allows us to understand the scale of low-value medical practices, measure practice change following specific interventions and prioritise policy decisions. To date, almost all research examining health care through the low-value lens has focused on medical services (tests and procedures) rather than on prescribing. The protocol described herein outlines a program of research funded by Australia’s National Health and Medical Research Council to select and quantify low-value prescribing practices within Australian routinely collected health data.

**Methods:**

We start by describing our process for identifying and cataloguing international low-value prescribing practices. We then outline our approach to translate these prescribing practices into indicators that can be applied to Australian routinely collected health data. Next, we detail methods of using Australian health data to quantify these prescribing practices (e.g. prevalence of low-value prescribing and related costs) and their downstream health consequences. We have approval from the necessary Australian state and commonwealth human research ethics and data access committees to undertake this work.

**Discussion:**

The lack of systematic and transparent approaches to quantification of low-value practices in routinely collected data has been noted in recent reviews. Here, we present a methodology applied in the Australian context with the aim of demonstrating principles that can be applied across jurisdictions in order to harmonise international efforts to measure low-value prescribing. The outcomes of this research will be submitted to international peer-reviewed journals. Results will also be presented at national and international pharmacoepidemiology and health policy forums such that other jurisdictions have guidance to adapt this methodology.

**Electronic supplementary material:**

The online version of this article (doi:10.1186/s13012-017-0585-9) contains supplementary material, which is available to authorized users.

## Background

There is a growing recognition in health care that less may actually be more and that safety and effectiveness may be compromised in the face of ‘too much medicine’ [1]. A recent US study examining regional variation in test ordering, procedures and prescribing found that up to 30% of practices are potentially harmful or wasteful [2]. Moreover, health care costs are spiralling due to the increasing volume of services and interventions for ageing populations and the introduction of new and emerging high-cost services and treatments. Low-value practices, including tests, procedures and prescribing that provide little or no benefit, may result in patient harm or wasted resources [3]. The process of identifying harmful or wasteful practices is not new, but the field has gathered renewed global momentum and publicity because of growing imperatives to maximise safety, effectiveness and benefits from health care investments [4]. There is a need to quantify nominated low-value practices at the population level in order to benchmark current activities, prioritise policy initiatives and measure changes resulting from policy interventions [5].

### Low-value prescribing practice nomination

There have been numerous attempts to systematically nominate low-value or inappropriate prescribing practices. However, in many circumstances, a lack of provider and public consultation has led to feelings of top-down stewardship and rationing [6–8]. Physician-led, consensus-based guidelines such as the Beers and STOPP criteria [9, 10] have also been developed to identify potentially inappropriate prescribing practices, but these tend to focus on specific populations (such as the elderly). Recent campaigns conducted through the lens of ‘low-value care’ have coalesced into the creation of specialty-specific, national lists of low-value practices that are not necessarily restricted to specific sub-populations [3]. Since 2007, the UK’s National Institute of Health and Care Excellence (NICE) has curated the ‘do not do’ guidance in association with the Cochrane Collaboration [6]. Similarly, the Choosing Wisely campaign is a grassroots physician-led program that was launched formally in 2012 in the USA and has since spread to Canada, Australia, Japan and Europe [11]. This program aspires to be a transparent and consistent process of speciality-wide consultation, in collaboration with patient representatives to nominate specialty-based ‘top 5 lists’ of low-value practices based on evidence [12] and shared values [7]. There are a number of other similar current initiatives detailing low-value practices within medicine such as the Royal Australasian College of Physicians’ EVOLVE program, the British Medical Journal’s Too Much Medicine series and the Journal of the American Medical Association’s Less is More series [13–15].

### Low-value prescribing practice quantification

The science of measuring low-value care is in its infancy. At the population level, quantification aims to determine the frequency of low-value practices as well as variation (geographical or by other factors), changes over time, associations with patient and provider characteristics and downstream health consequences and costs [5]. Recent advances in analytical methods and access to routinely collected health data have created possibilities for quantifying low-value care at the population level [16–18]. However, a major challenge is that many nominated practices and downstream consequences are finely nuanced and identify specific populations and clinical contexts that may not be recorded within routinely collected data [8].

There have been efforts to quantify harmful or wasteful prescribing using routinely collected data prior to the low-value movement [19], but attempts to systematically review the historical literature are challenging because of the lack of common key words and MeSH terms. Systematic reviews of pharmacoepidemiological studies undertaken using Australian and Nordic routine data collections found that studies quantifying harmful and/or wasteful practices were in the minority [20, 21]. Some examples include measuring the extent of potentially inappropriate medication use in the elderly [22, 23] and consensus-based lists of inappropriate psychotropic prescribing [24]. Following the evolution of low-value lists, there have been some efforts to benchmark low-value practice frequency and quantify the impact of the Choosing Wisely campaign using routine data collections. A recent systematic review of low-value measures identified a lack of transparency in translating practices into indicators and the need to validate any measures that are developed [25]. Studies to date have also tended to focus on low-value tests and procedures rather than on prescribing [26–28]. These studies use varying quantification approaches, thus limiting direct comparisons, and so, there is a need to standardise this methodology to enhance the global effort.

Therefore, the objectives of this research program are to detail methods for:

- Cataloguing nominated low-value prescribing practices from low-value lists published internationally. This process is ongoing and will be updated to incorporate additional practices appearing on international lists.
- Translating low-value prescribing practices into indicators that can be applied to Australian routine data collections.
- Quantifying the extent of low-value prescribing practices in Australia.
- Quantifying the downstream consequences of low-value prescribing practices in Australia.

The principles outlined in this protocol paper are generalisable to research in other jurisdictions attempting to quantify low-value prescribing practices in their routine data collections.

## Methods

### Cataloguing low-value prescribing practices

We are creating a catalogue of low-value prescribing practices by extracting nominated practices from international Choosing Wisely lists. Table 1 summarises the number of prescribing practices within each list as of August 2016 (full list of all practices available on request from authors). Collectively, these Choosing Wisely lists contain 189 individual prescribing practices representing 22% of all medical practices within these lists. We will update our catalogue as these lists continue to grow and other countries become engaged with the Choosing Wisely campaign.

**Table 1:** Number of prescribing practices by Choosing Wisely list as of August 2016

List	Total number practices within list^a^	Number (%) prescribing practices within list^a^
Choosing Wisely USA [64]	421	86 (20%)
Choosing Wisely Canada [65]	176	41 (23%)
Choosing Wisely Italy [66]	111	20 (18%)
Choosing Wisely Australia [67]	93	33 (35%)
Choosing Wisely International top 10 list [54]	10	5 (50%)
Choosing Wisely Netherlands [68]	35	2 (6%)
Choosing Wisely Switzerland [69]	10	2 (20%)

^a^Prescribing practices may be represented more than once within and between lists

#### Duplication of practices within and between lists

After identifying specific practices from the lists, we will break them into component parts: medication(s), indication(s), population(s) of intended use and other specifications. This assists with subsequent translation to indicators as part of the measurement process (described below). After grouping practices according to these components, we will identify similarities both within (when the practice was nominated by two different specialty groups on the same jurisdictional list) and between jurisdictional lists. In some cases, the practices will be an exact duplication. For example, within the Choosing Wisely USA list, ‘Don’t use antipsychotics as first choice to treat behavioural and psychological symptoms of dementia’ is specified using identical wording within both the American Society for Geriatrics and the American Psychiatric Association top 5 lists. In other instances, lists will nominate the same practice but use different wording. We also anticipate that similar practices may only have some common components, often meaning that they are broader or narrower definitions of a low-value practice.

#### Defining broader and narrower definitions of low-value practices

**Fig. 1:**
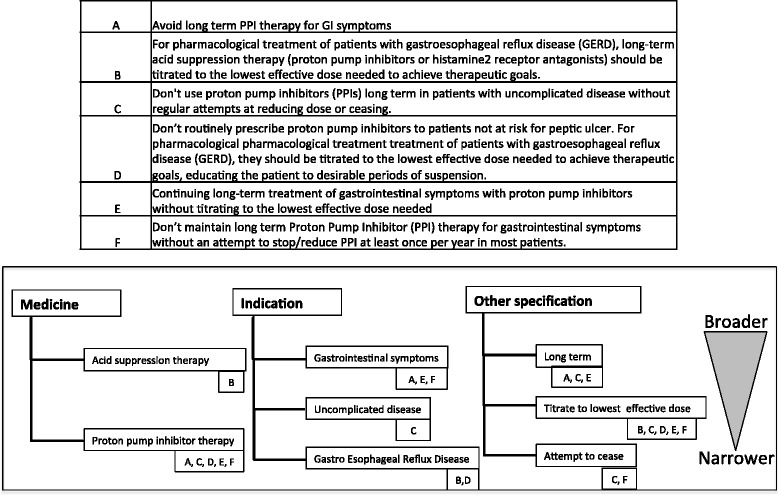
Example of categorising prescribing practices into broader and narrower definitions

Schwartz et al. [18] recognised that definitions of individual low-value practices vary within and across lists, meaning that broader and narrower definitions of similar practices may be measured to determine a range over which similar practices occur. The narrowest definition likely captures the most certain instances of low-value care but may exclude some cases classified as low value by other similar practices. In contrast, the broadest likely captures all instances but may also include some instances of care defined as appropriate by other similar practices. Where possible, we will identify broader and narrower definitions within lists of similar prescribing practices by identifying increasingly narrower definitions within the medicine component of the practice: medicine class, subclass or individual medicine. Within the indication component, we will specify organ-level disorders (broader) versus specific diagnoses (narrower). We will also identify prescribing practices with the same medicine and indication that specify subpopulations (e.g. by specifying an age range) and others that add caveats (within the ‘other specifications’ component) such as ‘first-line treatment’. For groups of practices with shared components, we will order the components of each prescribing practice according to whether they are broader or narrower than the corresponding components of other practices within the group (illustrated in Fig. 1).

### Translating low-value prescribing practices into indicators applied to Australian routine data collections

In this section, we first detail the study setting and the core characteristics of Australia’s routinely collected data as they pertain to the development of prescribing indicators and quantification of low-value prescribing practices. Second, we will detail the methods we will use to translate low-value practices into indicators applied to these data collections.

### Australia’s health care setting

Australia has a publicly funded universal health care system entitling all Australian citizens and permanent residents to a range of subsidised health services. This includes free treatment in public hospitals (funded jointly by the commonwealth and state/territory governments) and subsidised treatment in private hospitals (funded jointly by the commonwealth and private health insurance). It also includes a range of subsidised outpatient services including consultations with medical and selected health care professionals (funded by the commonwealth’s Medicare Benefits Scheme (MBS)) and medicines prescribed in the community and private hospitals and on discharge from some public hospitals (funded by the commonwealth’s Pharmaceutical Benefits Scheme (PBS); medicines prescribed to public hospital inpatients are covered primarily by hospital budgets). In addition, the Australian Government Department of Veterans’ Affairs (DVA) funds the health care of eligible veterans, war widows and widowers and their dependants. DVA clients are eligible to receive all health services and pharmaceuticals accessible to the general Australian population and, depending on their level of entitlement, additional DVA-approved services and pharmaceutical items. Historically, DVA data have been the primary data source for pharmacoepidemiological research in Australia as they are a sole payer and maintains custodianship over a wide variety of routine data collections including dispensing and medical service claims and hospitalisations. 

### Australia’s routine data collections

We restrict our commentary in this section to data collections (either stand-alone or linked to each other) that have the capacity to track individual patients over time. These collections are summarised in Table 2.

**Table 2:** Australian datasets (stand-alone or linked) available to quantify low-value prescribing practices

Dataset	Variables of interest
Medicines prescribed in the community	
Dispensing Dataset: Pharmaceutical Benefits Scheme (PBS) dispensing claims. Available 2005 to current Custodian: Australian Government Department of Human Services	Item number, date of prescribing and dispensing service, patient co-payment, cost to government, patient demographics (age, gender, location of residence mapped to Socioeconomic Index and Remoteness classifications), provider location (also mapped), fact of death
Prescribing Dataset: MedicineInsight. Available 2010 to current Custodian: NPS MedicineWise	Prescribed medicines (date of prescribing; prescribed daily dose; indication for prescribing), test ordering (date of request, results), patient demographics (age, gender, location of residence mapped to Socioeconomic Index and Remoteness classifications), patient past medical and family history, referrals, management plans, immunisations, provider location (also mapped)
Datasets to which PBS dispensing data will be linked	
Tests, procedures and outpatient consultations Dataset: Medical Benefits Schedule (MBS) service claims. Available 2004 to 2013 Custodian: Australian Government Department of Veterans Affairs	Item number, date of service, scheduled fee, provider charge, benefits paid, patient co-payment, patient demographics (age, gender, location of residence mapped to Socioeconomic Index and Remoteness classifications), provider location (also mapped)
Hospitalisations Dataset: NSW Admitted Patient Data Collection Available July 2000 to current Custodian: Director, Demand and Performance Evaluation Branch, NSW Department of Health	Diagnostic and procedure codes, diagnosis-related groups (admission costs), hospital type, separation date and status, geographic location of patient residence and hospital; patient demographics (age, gender, place of residence)
Emergency department visits Dataset: NSW Emergency Department Data Collection. Available 2005 to current Custodian: Director, Demand and Performance Evaluation Branch, NSW Department of Health	Triage category, diagnostic codes; mode of arrival; hospital type, geographic location of patient residence and hospital, separation date and status, patient demographics (age, gender, place of residence)
Cancer notifications Dataset: NSW Central Cancer Registry. Available 1994 to current Custodian: Central Cancer Registry NSW	Date of new cancer diagnosis, type of cancer, stage at diagnosis
Death data Dataset: Registry of Births, Deaths and Marriages NSW. Available 1994 to current Custodian: Registrar of Births, Deaths and Marriages NSW	Cause of death, place of death, date of death, decedent demographics (age, gender, place of usual residence)

There are two main sources of medicine data: (1) population-based pharmaceutical claims (payment) data captured after a prescribed medicine has been dispensed and subsidised under the PBS and (2) prescribing data captured at the point of care.

#### Pharmaceutical Benefits Scheme data

PBS claims will be the primary data source for the research program outlined in this protocol. Using dispensing claims data, we have the capacity to estimate temporal trends in dispensing as well as patterns of exposure (e.g. initiation, duration of use, dose and discontinuation) at the person level. PBS dispensing claims data also contain demographic details about the patient, such as age, gender and geographic location of residence, and also have the capacity to track prescriber behaviour through a unique prescriber identification number, although there is limited additional information about prescriber characteristics beyond their specialty of practice. PBS claims do not contain information such as the prescribed daily dose of a medicine or the indication for which a medication is prescribed. These features of PBS claims data are typical of dispensing claims data worldwide [29].

#### Prescribing data

Currently, Australia does not have any population-based, routinely collected prescribing datasets. Rather, data can be obtained and complied from individual community medical practices or public hospitals. Most general practices in Australia use electronic prescribing platforms that have the capacity to capture prescribing of PBS-listed and other medicines, but this is not necessarily the case in specialty practice [30, 31]. Medicines prescribed to public hospital inpatients are funded primarily by hospital budgets at the state level, and electronic prescribing systems have become increasingly commonplace in Australian public hospitals. Significant challenges currently exist in Australia in terms of unifying prescribing data across more than one hospital network and multiple community practices, due in part to the high degree of variability in software used and local governance arrangements. In addition, the quality and comprehensiveness of prescribing data is highly variable and dependent on end-user inputs.

However, one of the most promising prescribing datasets that has the potential for use in our study is from the MedicineInsight initiative [32], an Australian Government-funded programme that aims to increase the understanding of prescribing behaviour in Australian general practice. Comprising approximately 500 practices throughout Australia, it is the first large-scale, longitudinal general practice data program in Australia that extracts de-identified patient health records from existing general practice software. Compared with PBS dispensing data, these data contain greater depth of clinical information about the practice, the prescriber and the patient, as well as the clinical indication(s) for which the medicine is prescribed, tests are ordered and referrals are made [33]. However, because this is a relatively new program and general practice recruitment is ongoing, it is unclear how nationally-representative the data are currently.

#### Prescribed medicine data linked to other routinely collected datasets

We will utilise a range of population-based data comprising PBS dispensing claims linked to other routine collections held by the Commonwealth of Australia (Medicare service claims) and the individual states (hospitalisations, emergency department visits, cancer notification, fact and cause of death data) to implement our proposed research. For the purposes of the specific quantification, the example outlined below will use the DVA linked dataset, a data source that has been used extensively in Australian pharmacoepidemiological studies [20]. However, for future work quantifying other prescribing practices, we may use other linked datasets. Table 2 outlines the population-based collections that have been linked to dispensing claims data within the DVA dataset. The advantage of such linked collections is that they provide a more detailed picture of an individual’s health that can be helpful when attempting to isolate low-value practices. Linked data also enhance the capacity to establish the downstream consequences of low-value care and to account for confounders when performing outcome studies. While it is technically feasible to link prescribed medicine data at the individual level to other routine data collections, currently it will only yield data for a (non-representative or biased) sample of hospital or community settings in Australia. Efforts to link pathology results data to pharmaceutical dispensing claims on a national level are limited in Australia due to the multitude of pathology services, and as with integrating hospital prescribing nationally, there is a high degree of variability in software used and local governance arrangements.

### Indicator development and application

We will construct indicators by matching the component parts of prescribing practices to variables within routinely collected data. The collection of variables corresponding to a prescribing practice will represent the practice indicator. For the purposes of illustration, we have selected nine practices identified from the Choosing Wisely lists (Table 3). These practices represent the broadest definitions within their groups of similar practices. Practices that are similar but narrower are listed in Additional file 1: Table S1. In the simplest case, the components of the practice ‘don’t prescribe benzodiazepines to elderly people’ are benzodiazepines (medicine) and elderly people (population). Medicine and age are both variables within pharmaceutical dispensing claims data, and so, the indicator would consist of exposure to benzodiazepines and a defined age group and this practice could be measured in stand-alone PBS data.

**Table 3:** Low-value prescribing practice case examples and Choosing Wisely list origin

Prescribing practice example number	Practice	Choosing Wisely list
1	Avoid prescribing antibiotics for upper respiratory infections	USA
2	Don’t use benzodiazepines in the elderly	International
3	Avoid long-term PPI therapy for GI symptoms	International
4	Avoid antipsychotics for dementia	International
5	Do not use antibiotics in asymptomatic bacteriuria	Australia
6	Don’t recommend the regular use of oral non-steroidal anti-inflammatory medicines (NSAIDs) in older people	Australia
7	Don’t prescribe testosterone therapy unless there is evidence of proven testosterone deficiency	Australia
8	Don’t initiate and continue medicines for primary prevention in individuals who have a limited life expectancy	Australia
9	Don’t routinely prescribe two or more antipsychotic medications concurrently	USA

When we are unable to directly translate prescribing practices into indicators, as they contain components that do not have equivalent variables within existing datasets, we will create proxy measures from existing variables. For instance, the practice ‘avoid antipsychotics in dementia’ is comprised of antipsychotics (medicines) and dementia (indication). If attempting to quantify this within dispensing claims data alone, where indication for prescribing and information on co-morbidities are generally not available, we will create proxy markers for disease states (such as the prior dispensing of an anti-dementia medication). Similarly, for the practice ‘Don’t routinely prescribe two or more antipsychotic medications concurrently’, we will use a proxy for medication concomitance within dispensing claims data as the intended duration of exposure following a medication dispensing is not known. The uncertainty introduced by these proxies can be somewhat mitigated if dispensing claims are linked to other data sources such as hospitalisation data, which contain hospitalisation-related diagnostic codes. While the degree of uncertainty introduced by these proxy measures is difficult to quantify, we will perform sensitivity analyses, such as varying the boundaries of decision rules within these proxy variables to observe the magnitude of the effect on practice quantification. Prescribing data can also be used if an indication is required and cannot be inferred from dispensing claims or hospitalisations. However, as previously discussed, the quality of diagnostic information is end-user dependent and we aim to focus on dispensing claims for this Australian analysis as the generalisability of findings from existing Australian prescribing data are unclear. Furthermore, we do not currently have access to dispensing claims linked to pathology data. However, in the interests of this protocol being adaptable to an international audience, we have included prescribing and pathology data at the stage of indicator translation. Table 4 illustrates how the nine exemplar prescribing practices (Table 3) can be translated into their corresponding indicators in Australian data.

**Table 4:** Dataset requirements for quantifying exemplar low-value prescribing practice examples

Indicator components	Prescribing practice								
	1. Antibiotics in URTIs	2. Benzodiazepines in elderly	3. Long-term PPIs	4. Antipsychotics in dementia	5. Antibiotics in bacteriuria	6. NSAIDs in elderly	7. Testosterone	8. Primary prevention in limited life	9. Antipsychotic polypharmacy
Dispensing data									
Patient demographics^a^		X		X		X		X	
Initiation (date of dispensing)	X	X	X	X	X	X	X	X	X
Discontinuation^b^			X						
Concomitant use^b^									X
Switching^b^									
Comorbidity^b^				X				X	
Fact of death								X	
Prescribing data									
Patient demographics^a^		X				X			
Initiation	X	X	X	X	X	X	X	X	X
Discontinuation			X						
Concomitant use									X
Switching									
Indication stated by prescriber	X		X	X	X		X	X	
Test ordering					X		X		
Comorbidities				X				X	
Hospitalisation data									
Co-morbidity (ICD-10 code)				X			X	X	
Pathology data									
Test results					X		X		
Death data									
Date of death								X	
Cancer data									
Cancer diagnosis								X	

Prescribing practices correspond with exemplar practices listed in Table 3. An ‘X’ marks where a component indicator corresponds to a component within a prescribing practice

^a^Includes age, gender, geographic location of residence

^b^Proxy measures (i.e. the result of decision rules applied to existing variables)

There may often be several approaches to quantifying the same practice that vary in accuracy and precision by using different indicators comprised of a range of different variables, potentially from different data sources. When adapting these methods to other jurisdictions, the most appropriate approach depends on local data access, coverage and analytical expertise.

### Quantifying the extent of a low-value prescribing practice in Australia

Our research program will undertake a series of observational cohort studies to quantify the extent of low-value prescribing in Australia. In this section, we describe specific quantification methodology for one low-value prescribing practice, ‘Don’t routinely prescribe two or more antipsychotic medications concurrently’, specified within the USA Choosing Wisely list and hereafter referred to as antipsychotic polypharmacy. The general approach outlined here will apply to all of the low-value prescribing practices detailed in this protocol when we utilise PBS claims linked with other routine collections.

#### Cohort definition

We will use stand-alone Pharmaceutical Benefits Scheme (PBS) dispensing claims from 1 March 2005 to the most contemporary available (currently January 2017) to perform our analysis [34] (Table 2). We will identify people dispensed antipsychotics (WHO Anatomical Therapeutic Classification codes NO5A) and define antipsychotic concomitance as overlapping courses of two or more non-like antipsychotics for 60 days or more. However, we will also perform sensitivity analyses using 14- and 90-day cutoffs. PBS dispensing claims contain only the date medicines are dispensed, and so, to identify courses of antipsychotics, we will first estimate the duration of exposure (in days) following a single dispensing of each antipsychotic medicine, hereafter referred to as the estimated period of exposure (EPE). For each antipsychotic, we will measure the intervals between dispensings of the same medicine in individuals who have more than one dispensing of that medicine over the study period. The EPE for a medicine will then be calculated as the number of days in which 75% of people have received a subsequent dispensing of the same medicine. A course of a given antipsychotic medicine will be defined as the period in which there are serial dispensings of the same medicine and the EPEs of these dispensings overlap. This method has been applied previously to psychotropics and accounts for variability in dispensing due to adherence, dose changes and seasonality [35, 36].

#### Practice quantification

We will quantify the extent of and variability in antipsychotic polypharmacy. Specifically, we will determine:

- The number of patients experiencing antipsychotic polypharmacy expressed as a proportion of all people treated with antipsychotics. We will calculate the number of episodes of antipsychotic polypharmacy as well as the number (mean, median, interquartile range (IQR) and maximum and minimum) of episodes per patient. We will describe this cohort according to age, gender, location of residence, rurality and socioeconomic stratum.
- The number of prescribers delivering low-value prescribing expressed as a proportion of the total number of prescribers in the study cohort. We will also calculate the number (means, medians, interquartile ranges and maxima and minima) of episodes of antipsychotic polypharmacy per prescriber and whether antipsychotic polypharmacy was initiated or continued by that prescriber. We will then describe these prescribers according to available prescriber variables. Within the PBS dispensing claims, this is limited to speciality, but in other datasets, in particular prescribing data, additional variables include number of years in practice and rurality and socioeconomic characteristics of practice location.
- Variation in low-value care. We will examine the variation in antipsychotic polypharmacy by stratifying analyses according to prescriber location (rurality and socioeconomic stratum of practice location) and patient characteristics (age, gender, location of residence, rurality and socioeconomic stratum) using established methodology [37, 38]. We will also use Poisson, negative binomial and/or multilevel regression models to determine the influence of these patient and provider characteristics on antipsychotic polypharmacy counts [39].
- Changes over time and following policy intervention. Analyses a. to c. will be annualised to demonstrate changes over time. We will calculate the prevalence and incidence of antipsychotic polypharmacy where *prevalent use* is defined as persons with at least one episode of antipsychotic polypharmacy within a given calendar year and *incident (new) use* is defined as persons with an episode of antipsychotic polypharmacy within a given calendar year and no episode of antipsychotic polypharmacy in the previous 12 months. Additionally, we will use interrupted time series analyses as previously applied to PBS dispensing claims [40, 41] to examine changes in antipsychotic polypharmacy before and after interventions that may impact on this practice. An example of such an intervention in Australia that will be investigated is the change in the prescribing restrictions for antipsychotics that had required telephone authority but moved to streamlined authority on 1 July 2007 [42]. With fewer barriers to prescribing, this may have increased antipsychotic polypharmacy. Given concerns about antipsychotic polypharmacy, this may prompt a review of prescribing authority policy. We will use an autoregressive integrated moving average (ARIMA) approach using the Box-Jenkins method which will be used to model subsequent time series while accounting for seasonal variability, as well as long-term trends and autocorrelation [43].

### Quantification of downstream consequences

Here, we start with a discussion regarding the general concepts involved in quantifying downstream consequences of low-value prescribing practices within routinely collected data and then return to detail how these are applied to quantify the downstream consequences of antipsychotic polypharmacy. Low-value prescribing practices may expose individuals to additional, unnecessary risks, and investigating the measurable harms of these practices is an important research endevour. Adverse drug events (ADEs) known to be associated with each practice will be identified from the literature, and valid indicators corresponding to these ADEs subsequent to an index dispensing will be identified within routinely collected health datasets. These may be medicine-based ADE indicators such as the dispensing of a medicine known to be associated with an ADE or diagnostic indicators, such as ICD-10 codes in hospitalisation data or the International Classification of Primary Care (second edition) classification (ICPC) system used in general practice. ADE [44] and disease state [45] indicators have been validated in Australian veterans’ affairs data. While disease state indicators were validated to measure co-morbidity, they also have clinical face validity as ADE markers. Generally, these ADE indicators have been shown to have good specificity but lower sensitivity when compared to the gold standards of record review and self-reported health surveys [44–46]. The other limitation is that they vary in how accurately they reflect the actual timing of onset of the ADE. Additional file 2: Table S2 identifies the potential ADEs associated with each prescribing practice example in Table 3, and Additional file 3: Table S3 details the potential medicine and diagnostic-based indicators of these adverse drug events.

Antipsychotic polypharmacy has been associated with increased risk of parkinsonism, dyslipidaemia, cardio and cerebrovascular disease, diabetes, cognitive impairment and sudden cardiac death (Additional file 2: Table S2) [47].Using the same study cohort and study period defined above, we will use prescription sequence analysis [44] to determine the association of antipsychotic polypharmacy with an incident dispensing (i.e. new dispensing with no dispensings in the 12 months prior) of these ADE medicine-based indicators. The crude sequence ratio will be calculated by dividing the number of people with an incident dispensing of an ADE indicator medicine in a 12-month post-exposure period (after the start of antipsychotic polypharmacy) with the number of people with incident dispensing of an ADE indicator medicine in a 12-month pre-exposure period (before the start of antipsychotic polypharmacy). We will perform sensitivity analyses, varying the length of pre- and post-exposure periods (3, 6 and 18 months). We will estimate the probability that the ADE medicine is dispensed first, in the absence of any causal relationship, adjusting for the null-effect sequence ratio which accounts for underlying changes in the incidence of dispensing of the ADE medicine (see [48] for details and formula). We will then calculate the adjusted sequence ratio (ASR) by dividing the crude by the null-effect ratio, and 95% confidence intervals will be calculated for this value. A signal is considered to be present when the lower limit of the 95% CI is one or more.

When dispensing claims data are linked to other health databases, a greater range of outcome variables are available. In these cases, cohort and case-control study designs with appropriate regression analyses [49] can be used to quantify the association between an index prescribing event and an ADE using an appropriate ADE indicator while adjusting for patient and prescriber factors. Choice of study design will depend on the known natural history of the ADE, its temporal relationship to the low-value practice exposure and the availability of a control group.

In this specific example, we will apply a self-controlled case series analysis to determine whether antipsychotic polypharmacy leads to increased risk of acute vascular disease. This will require linkage of PBS dispensing claims and hospitalisation data, and for this purpose, we have access to Department of Veterans Affairs (DVA) linked dataset (Table 2). Self-controlled case series analysis effectively measures incidence rate ratios of relatively acute ADEs while controlling for measured and unmeasured fixed patient confounders [50, 51]. We will use PBS dispensing claims linked to hospitalisation data for DVA beneficiaries from 1 January 2006 and 31 December 2015 but results will be reported from 1 January 2007 to allow for a 1-year look-back when identifying incident antipsychotic polypharmacy. Patients will be eligible for inclusion if they have hospital diagnostic codes related to acute ischemic heart disease (ICD-10: I20-24) and stroke (ICD-10: I60-I64) during the study period and if they had been full entitlement holders (eligible for all health services) for at least 12 months at the time of diagnosis. Incident antipsychotic polypharmacy (i.e. new episodes with no episodes in the 12 months prior) will be identified for these individuals during this period. The end of antipsychotic polypharmacy will be defined as one EPE after the last concomitant antipsychotic dispensing. As previously described [52] exposed person-time will be divided into 1, 2 to 4 and 5 to 12 weeks after the start of antipsychotic polypharmacy. A pre-exposure risk period will also be divided into 1, 2 to 4 and 5 to 12 weeks prior to the start of antipsychotic polypharmacy, and this will be considered separately from non-exposed time to account for confounding by indication (i.e. in the case that polypharmacy was initiated as a consequence of hospitalisation). Some individuals will have no exposure to antipsychotic polypharmacy for the entire study period but will be included in the analysis to contribute information about possible confounders. The count of outcomes in each of these exposure risk periods will be compared with the count of outcomes in non-exposed periods. Conditional Poisson regression will be used to calculate incidence rate ratios for the two primary outcomes (hospitalisation with ischemic heart disease or stroke) adjusting for age, gender and calendar year.

#### Incremental costs

We will use dispensing claims data to calculate the medication costs to the payer (government or private health care fund) and out-of-pocket costs to the patient for psychotropic polypharmacy and compare these costs to antipsychotic monotherapy. Excess hospitalisations with cardiovascular diseases and stroke will be extrapolated from incidence rate ratios, and excess costs will be calculated using length of stay and hospital diagnosis-related groups (DRGs) [53].

## Ethics

The Population Health Sciences Research Ethics Committee has granted approval for our overall program of research (Approval Numbers 2013/11/494 and 2014/06/539).

### Consent and privacy considerations

A combination of commonwealth, state, hospital and privately held data will be used as part of this program of work.

The use and disclosure of commonwealth and state data are governed under the Privacy Act 1988 and Health Records and Information Privacy (HRIP) Act 2003, respectively. We were able to access this data with a waiver of consent as under this legislation, it is not required if (1) it is impracticable to gain consent and (2) the use is in accordance with the section 95A guidelines (which provides a process to resolve the conflict that may arise between the public interest in privacy and the public interest in medical research).

We will minimise the risk to personal privacy by ensuring data are stored securely within the Centre for Big Data Research located at the University of New South Wales; only researchers involved in data analysis will have access to the data and that researchers do not have access to identified data or any means of re-identifying individuals within the data. Finally, all data will be presented in aggregated form only, and potentially identifiable information will not be published. We will suppress data with small cell sizes.

## Dissemination

The outcomes of this research will be submitted to international peer-reviewed journals; in particular general medical, health policy and pharmacoepidemiology journals. Furthermore, results will be presented at national and international pharmacoepidemiology, health policy and Choosing Wisely forums. Direct access to the data and analytical files to other individuals or authorities is not permitted without the express permission of the approving human research ethics committees and data custodians.

## Limitations

The potential limitations of this programme of research relate to low-value lists themselves and the application of these practices for quantification in routine data collections. The Choosing Wisely campaign has been criticised for the inclusion of low-impact, low-value practices, putatively because of conflict of interest with revenue streams for specific specialist groups [8]. However, this appears to be the exception rather than the rule [8], and one may argue that this is not likely to be the case for prescribing that typically does not generate income streams for physicians. The process of practice translation into indicators highlights limitations of using routine data collections to quantify prescribing practices such as inferences and things that cannot be easily measured. This is one consequence of lists that have been generated without measurement necessarily in mind and often results from lack of clinical information within routine data collections. This can also lead to substantial unmeasured confounding when attempting to identify associations between low-value prescribing practices and downstream ADEs. Using self-controlled study designs can help mitigate this unmeasured confounding. There is a need to validate more ADE indicators within Australian datasets using either medical record review or self-reported health surveys as gold standards. Issues such as data coverage and data access also limit generalisability of results.

## Discussion

The presence of prescribing practices in international Choosing Wisely initiatives clearly indicates there is cause for concern regarding low-value prescribing. However, to date there have been no systematic efforts to quantify these practices at a jurisdictional level. Furthermore, there have been no efforts to systematically determine the downstream consequences and associated costs of these low-value prescribing practices.

We noted similarities in prescribing practices within and between jurisdictional lists and that similar practices could be grouped (mostly according to medication and indication) during our first cataloguing process. Identifying groups of similar practices is one approach to identify priority areas for practice measurement: large groups of similar practices may represent shared areas of concern. They also represent opportunities to harmonise low-value practice definitions at jurisdictional and international levels by agreeing on a common list of practices to endorse. This would greatly assist quantification as within and between jurisdictional variability in low-value practice frequency could then be more easily measured and Choosing Wisely International has already started this process [54]. Until there is a common international list, identifying and quantifying broader and narrower definitions of similar practices allows a range over which similar practices occur to be identified. This method has been applied to quantifying tests and procedures [18]. However, compared to low-value tests and procedures, identifying the narrowest definitions of prescribing practices can be more challenging due to the greater and more varied number of components that prescribing practices are composed of.

As noted previously in our introduction, there are a number of other low-value lists such as the National Institute for Health and Care Excellence (NICE) ‘do not do’ list, the Royal Australasian College of Physicians EVOLVE project [11] and practices highlighted within *JAMA Internal Medicine's* and *BMJ's* ‘less is more’ and ‘too much medicine’ series, respectively. For the purposes of this protocol, we have focused exclusively on Choosing Wisely, but we intend on cataloguing all practices with other lists as they become available. It is important to note that many of the items identified within the Choosing Wisely lists are also present in these other lists.

Quantification efforts using routinely collected health data specifically assist in benchmarking population levels and costs of low-value practices as well as evaluating the impact of ensuing policy and practice decisions [5]. These quantification efforts may also directly facilitate behaviour change by providing methods of non-punitive audit and feedback to prescribers and patients [22].

Although Australian data has been used as an exemplar, it is our intent that these methods can be used by other jurisdictions as a standard framework to continue to prioritise and quantify prescribing practices at a jurisdictional level.

## Additional files

Additional file 1: Table S1. Low-value prescribing practice case examples with broadest (highlighted) and narrower definitions along with Choosing Wisely list origin. (DOCX 105 kb)

Additional file 2: Table S2. Adverse drug events related to prescribing practice case examples as identified from the literature [47, 50, 55–63]. (DOCX 76 kb)

Additional file 3: Table S3. Pharmacy- and hospitalisation-based indicators of adverse drug events/diseases states identified in Additional file 2: Table S2 [44, 45]. (DOCX 96 kb)
