# Supplementary material for: A methodological protocol for selecting and quantifying low-value prescribing practices in routinely collected data: an Australian case study
Source: Implement Sci. 2017 May 3;12:58. doi: 10.1186/s13012-017-0585-9 (PMC5415810; doi:10.1186/s13012-017-0585-9)
Supplement: Supplementary file 1 — Low-value prescribing practice case examples with broadest (highlighted) and narrower definitions along with Choosing Wisely list origin. (DOCX 105 kb) [file 13012_2017_585_MOESM1_ESM.docx]

**Table S1:** Low-value prescribing practice case examples with broadest (highlighted) and narrower definitions along with Choosing Wisely List origin

| **Prescribing practice example number** | **Definition** | **Practice** | **Choosing Wisely List** |
| --- | --- | --- | --- |
| 1 | Broadest | Avoid prescribing antibiotics for upper respiratory infections. | USA |
|  |  | Antibiotics should not be used for apparent viral respiratory illnesses (sinusitis, pharyngitis, bronchitis). | USA |
|  |  | Don’t routinely prescribe antibiotics for acute infections of the upper airways. | Italy |
|  |  | Prescribing antibiotics for uncomplicated upper respiratory tract infections. | Switzerland |
|  |  | Don’t use antibiotics for upper respiratory infections that are likely viral in origin, such as influenza-like illness, or self-limiting, such as sinus infections of less than seven days of duration. | Canada |
|  |  | Don't prescribe antibiotics for sinusitis. | International |
|  |  | Don't routinely prescribe antibiotics for acute mild-to-moderate sinusitis unless symptoms last for seven or more days, or symptoms worsen after initial clinical improvement. | USA |
|  |  | Avoid prescribing antibiotics in the emergency department for uncomplicated sinusitis. | USA |
| 2 | Broadest | Don't use benzodiazepines in the elderly. | International |
|  |  | Don't use benzodiazepines or other sedative-hypnotics in older adults as first choice for insomnia, agitation or delirium. | USA |
|  |  | Don’t use benzodiazepines or other sedative-hypnotics in older adults as first choice for insomnia. | Italy |
|  |  | Do not use benzodiazepines or other sedative-hypnotics in older adults as first choice for insomnia. | Canada |
|  |  | Don’t routinely prescribe benzodiazepines or Z-drugs in older adults as first choice for insomnia. Recommend to use them intermittently and to periodically reassess the clinical indication as well as any side effects. | Italy |
|  |  | Don’t use benzodiazepines or other sedative-hypnotics in older adults as first choice for insomnia, agitation or delirium. | Canada |
|  |  | Don’t use benzodiazepines and other sedative-hypnotics in older adults as first choice for insomnia, agitation or delirium. | Canada |
| 3 | Broadest | Avoid long-term PPI therapy for GI symptoms. | International |
|  |  | Continuing long-term treatment of gastrointestinal symptoms with proton pump inhibitors without titrating to the lowest effective dose needed. | Switzerland |
|  |  | Don’t maintain long-term Proton Pump Inhibitor (PPI) therapy for gastrointestinal symptoms without an attempt to stop/reduce PPI at least once per year in most patients. | Canada |
|  |  | For pharmacological treatment of patients with gastroesophageal reflux disease (GERD), long-term acid suppression therapy (proton pump inhibitors or histamine2 receptor antagonists) should be titrated to the lowest effective dose needed to achieve therapeutic goals. | USA |
|  |  | Don't use proton pump inhibitors (PPIs) long term in patients with uncomplicated disease without regular attempts at reducing dose or ceasing. | Australia |
|  |  | Don’t routinely prescribe proton pump inhibitors to patients not at risk for peptic ulcer. For pharmacological treatment of patients with gastroesophageal reflux disease (GERD), they should be titrated to the lowest effective dose needed to achieve therapeutic goals, educating the patient to desirable periods of suspension. | Italy |
| 4 | Broadest | Avoid antipsychotics for dementia. | International |
|  |  | Don't use antipsychotics as first choice to treat behavioural and psychological symptoms of dementia. | USA |
|  |  | Don't use antipsychotics as first choice to treat behavioural and psychological symptoms of dementia. | USA |
|  |  | Do not use antipsychotics as the first choice to treat behavioural and psychological symptoms of dementia | Australia |
|  |  | Don't use antipsychotics as first choice to treat behavioural and psychological symptoms of dementia. | Canada |
|  |  | Do not use antipsychotics as first choice to treat behavioural and psychological symptoms of dementia. | Canada |
|  |  | Don't prescribe antipsychotic medications for behavioural and psychological symptoms of dementia (BPSD) in individuals with dementia without an assessment for an underlying cause of the behaviour. | USA |
|  |  | Don’t use antipsychotics as a first choice to treat behavioural symptoms of dementia. Identifying and addressing causes of behaviour change can make treatment unnecessary. | Italy |
| 5 | Broadest | Do not use antibiotics in asymptomatic bacteriuria. | Australia |
|  |  | Don’t treat asymptomatic bacteriuria with antibiotics. | USA |
|  |  | Don’t prescribe antibiotics for asymptomatic bacteriuria (ASB) in non-pregnant patients. | Canada |
|  |  | Avoid antimicrobials for bacteriuria in elderly. | International |
|  |  | Don’t use antimicrobials to treat asymptomatic bacteriuria in the elderly. | Canada |
|  |  | Don’t use antimicrobials to treat bacteriuria in older adults unless specific urinary tract symptoms are present. | Canada |
|  |  | Don't use antimicrobials to treat bacteriuria in older adults unless specific urinary tract symptoms are present. | USA |
|  |  | Do not treat with antibiotics bacteriuria in the elderly, in the absence of urinary symptoms. | Italy |
| 6 | Broadest | Don’t recommend the regular use of oral non-steroidal anti-inflammatory medicines (NSAIDs) in older people | Australia |
|  |  | Avoid nonsteroidal anti-inflammatory drugs (NSAIDS) in individuals with hypertension or heart failure or CKD of all causes, including diabetes. | USA |
|  |  | Don’t prescribe nonsteroidal anti-inflammatory drugs (NSAIDS) in individuals with hypertension or heart failure or CKD of all causes, including diabetes. | Canada |
| 7* |  | Don’t prescribe testosterone therapy unless there is evidence of proven testosterone deficiency | Australia |
|  |  | Don’t prescribe testosterone therapy unless there is evidence of proven testosterone deficiency | Canada |
|  |  | Don't prescribe testosterone to men with erectile dysfunction who have normal testosterone levels. | USA |
| 8 | Broadest | Don’t initiate and continue medicines for primary prevention in individuals who have a limited life expectancy | Australia |
|  |  | Don't routinely prescribe lipid-lowering medications in individuals with a limited life expectancy. | USA |
| 9 | Broadest | Don't routinely prescribe two or more antipsychotic medications concurrently. | USA |
|  |  | Do not routinely prescribe high-dose or combination antipsychotic treatment strategies in the treatment of schizophrenia. | Canada |

*Broadest could not be determined.
