# Supplementary material for: A methodological protocol for selecting and quantifying low-value prescribing practices in routinely collected data: an Australian case study
Source: Implement Sci. 2017 May 3;12:58. doi: 10.1186/s13012-017-0585-9 (PMC5415810; doi:10.1186/s13012-017-0585-9)
Supplement: Supplementary file 2 — Adverse drug events related to prescribing practice case examples as identified from the literature [47, 50, 55–63]. (DOCX 76 kb) [file 13012_2017_585_MOESM2_ESM.docx]

**Table S2:** Adverse drug events related to prescribing practice case examples as identified from the literature

| **Prescribing practice example number** | **Prescribing practice** | **Adverse Drug Event** |
| --- | --- | --- |
| 1 | Don’t routinely prescribe antibiotics for acute infections of the upper airways. | Antimicrobial resistance, Clostridium difficile infection (55) |
| 2 | Don't use benzodiazepines in the elderly | Fall, hip fracture, cognitive decline, substance use disorder, overdose, hospitalisation, death (56) |
| 3 | Avoid long-term proton pump inhibitor (PPI) therapy for gastrointestinal symptoms | Pneumonia (50), Clostridium difficile infection (57), hip fracture (58) |
| 4 | Avoid antipsychotics for dementia | Stroke, acute coronary syndrome, hyperlipidaemia, diabetes, sudden cardiac death, fall, hip fracture, cognitive decline (59) |
| 5 | Avoid antimicrobials for bacteriuria in elderly | Antimicrobial resistance, Clostridium difficile infection (60) |
| 6 | Don’t recommend the regular use of oral non-steroidal anti-inflammatory medicines (NSAIDs) in older people | Heart failure, acute coronary syndrome, stroke, death, hypertension, hospitalisation (61), renal failure (62) |
| 7 | Don’t prescribe testosterone therapy unless there is evidence of proven testosterone deficiency | Unclear (63) |
| 8 | Don’t initiate and continue medicines for primary prevention in individuals who have a limited life expectancy | N/A |
| 9 | Don't routinely prescribe two or more antipsychotic medications concurrently. | Stroke, acute coronary syndrome, hyperlipidaemia, diabetes, sudden cardiac death, fall, hip fracture, cognitive decline (47) |
|  |  |  |
