# Supplementary material for: A methodological protocol for selecting and quantifying low-value prescribing practices in routinely collected data: an Australian case study
Source: Implement Sci. 2017 May 3;12:58. doi: 10.1186/s13012-017-0585-9 (PMC5415810; doi:10.1186/s13012-017-0585-9)
Supplement: Supplementary file 3 — Pharmacy- and hospitalisation-based indicators of adverse drug events/diseases states identified in Additional file 2: Table S2 [44, 45]. (DOCX 96 kb) [file 13012_2017_585_MOESM3_ESM.docx]

**Table S3**: Pharmacy and hospitalisation based indicators of adverse drug events/diseases states identified in Table S2

| **Adverse drug event/disease state** | **Medicine based indicator** | **Diagnostic indicator** |
| --- | --- | --- |
| Acute coronary syndrome | None | Hospitalisation related to acute ischemic heart disease (ICD-10: I20-24) |
| Antimicrobial resistance | None | None |
| Clostridium difficile infection | None | Hospitalisation with Clostridium difficile colitis (ICD-10: A04.7) |
| Death | Death recorded in PBS data | None |
| Dementia (or cognitive decline) | Dementia: Anti-dementia medication (ATC:NO6D)^$^ | Hospitalisation with dementia (ICD-10: F02) |
| Diabetes | Oral hypoglycaemic (ATC A10B) ^$^*, insulin (ATC A10A)* | Hospitalisation with diabetes type 2 (ICD-10: E11) |
| Drug dependence | None | Hospitalisation with mental disorder related to sedatives, hypnotics, anxiolytics (ICD-10: F13) |
| Fall | None | Hospitalisation with fall (ICD-10: W00-W19) |
| Heart failure | loop diuretics (ATC: C03CA) ^$^, ACE (ATC: C09A and B) ^$^ and angiotensin II inhibitors (ATC: C09C and D) ^$^ | Hospitalisation with congestive cardiac failure (ICD-10: I50) |
| Hip fracture | None | Hospitalisation with hip fracture (ICD-10: S720, S721) |
| Hospitalisation | None | Any hospitalisation |
| Hyperlipidaemia | Statin (ATC: C10AA, C10B) ^$^* | None |
| Hypertension | Thiazides, potassium-sparing agents, combination antihypertensives, other antihypertensives (e.g. clonidine, hydralazine) (ATC: C02A to L) ^$^ | None |
| Overdose | None | Hospitalisation with overdose (ICD-10: T36-T50) |
| Parkinsonism | Levodopa (ATC: NO4BA) | Hospitalisation with parkinsonism (ICD-10: G20) |
| Pneumonia | None | Hospitalisation with pneumonia (ICD-10: J12, J13, J14, J15, J16, J17, J18) |
| Renal failure | End stage renal disease: alpha erythropoietin (ATC: B03XA01), calciferol (ATC: A11CC05), calcitriol (ATC: A11CC04), sevelamer (ATC: V03AE02) $ | Hospitalisation with renal failure* (ICD-10: N17, N18, N19) |
| Stroke | None | Hospitalisation with stroke* (ICD-10: I60-I64) |
| Sudden cardiac death | None | None |

*Validated as adverse drug event marker in Australian data (44). $ Validated as disease state marker in Australian data (45).
